# Causal inference study on the effect of exogenous insulin on the risk of osteoarthritis

**DOI:** 10.1097/MD.0000000000045780

**Published:** 2025-12-12

**Authors:** Jinzhe Zheng, Wei Li, Gensheng Zhang, Yangyang Liu, Jichao Liu

**Affiliations:** a Department of Orthopedics, 3201 Hospital of Xi’an Jiaotong University Health Science Center, Hanzhong City, Shaanxi Province, China; b Spine Department, 2 Hanzhong Central Hospital, Hanzhong City, Shaanxi Province, China.

**Keywords:** causal inference, exogenous insulin, Mendelian randomization, osteoarthritis

## Abstract

The study explores the previously unexamined link between exogenous insulin treatment for diabetes mellitus and its potential to alter the risk of osteoarthritis (OA) using Mendelian randomization (MR) to investigate a possible causal relationship. By employing a variety of two-sample MR methodologies, including instrumental variable weighting (IVW), weighted median, MR-Egger, weighted mode, simple mode, and conducting multivariate MR (MVMR) Analysis, this research aims to assess the direct effects of exogenous insulin on OA, while also considering the mediating roles of diabetes and smoking status. Our findings through the IVW model reveal a significant causal association, indicating that exogenous insulin use is linked to a decreased risk of OA, evidenced by an odds ratio (OR) of 0.00029 (95% CI: 0–0.694, *P* = .040158), with no causal effect identified from OA towards exogenous insulin in reverse MR analysis. Further mediation analysis highlights diabetes and smoking as significant mediators of insulin’s effect on OA, with diabetes and smoking respectively accounting for 74.26%, 70.11%, and 43.34% of the mediation effect through various indicators. Consequently, this MR study provides substantial evidence of a causal relationship between exogenous insulin use and a reduced OA risk, emphasizing the importance of holistic management for diabetic patients to carefully weigh the benefits of insulin therapy against its potential OA risks.

## 1. Introduction

Osteoarthritis (OA), a leading cause of morbidity and disability worldwide, presents a growing challenge, particularly in aging populations.^[1,2]^ Understanding its multifaceted etiology, including modifiable risk factors, is crucial for addressing its rising prevalence. OA’s pathogenesis is attributed to factors such as aging,^[3]^ genetic predispositions,^[4]^ joint injuries, abnormal joint loading,^[5]^ and metabolic aspects.^[6]^

Recent evidence underscores the significant influence of metabolic syndrome components obesity, hypertension, and diabetes mellitus (DM) on OA’s development.^[7,8]^ Notably, hyperglycemia, DM’s hallmark, is increasingly acknowledged as a key contributor to OA progression through disruptions in glucose metabolism, vascular changes, and inflammatory pathways, adversely impacting joint health.^[9–11]^

While the link between hyperglycemia and OA is becoming clearer, the specific effects of exogenous insulin therapy in OA’s pathogenesis require further exploration. Our study seeks to address this gap by leveraging the Mendelian randomization (MR)^[12]^ approach to elucidate the causal relationship between exogenous insulin use and OA. MR’s unique ability to reduce confounding and reverse causation offers a robust alternative to conventional observational studies.^[13]^ Furthermore, by incorporating reverse MR analysis and examining additional risk factors such as smoking and diabetes, we conduct a comprehensive Multivariable MR analysis and mediation effect analysis to ensure a thorough investigation of the direct and indirect effects of exogenous insulin on OA.

## 2. Materials and methods

### 2.1. Study reporting guidelines and study design

**Figure 1.**
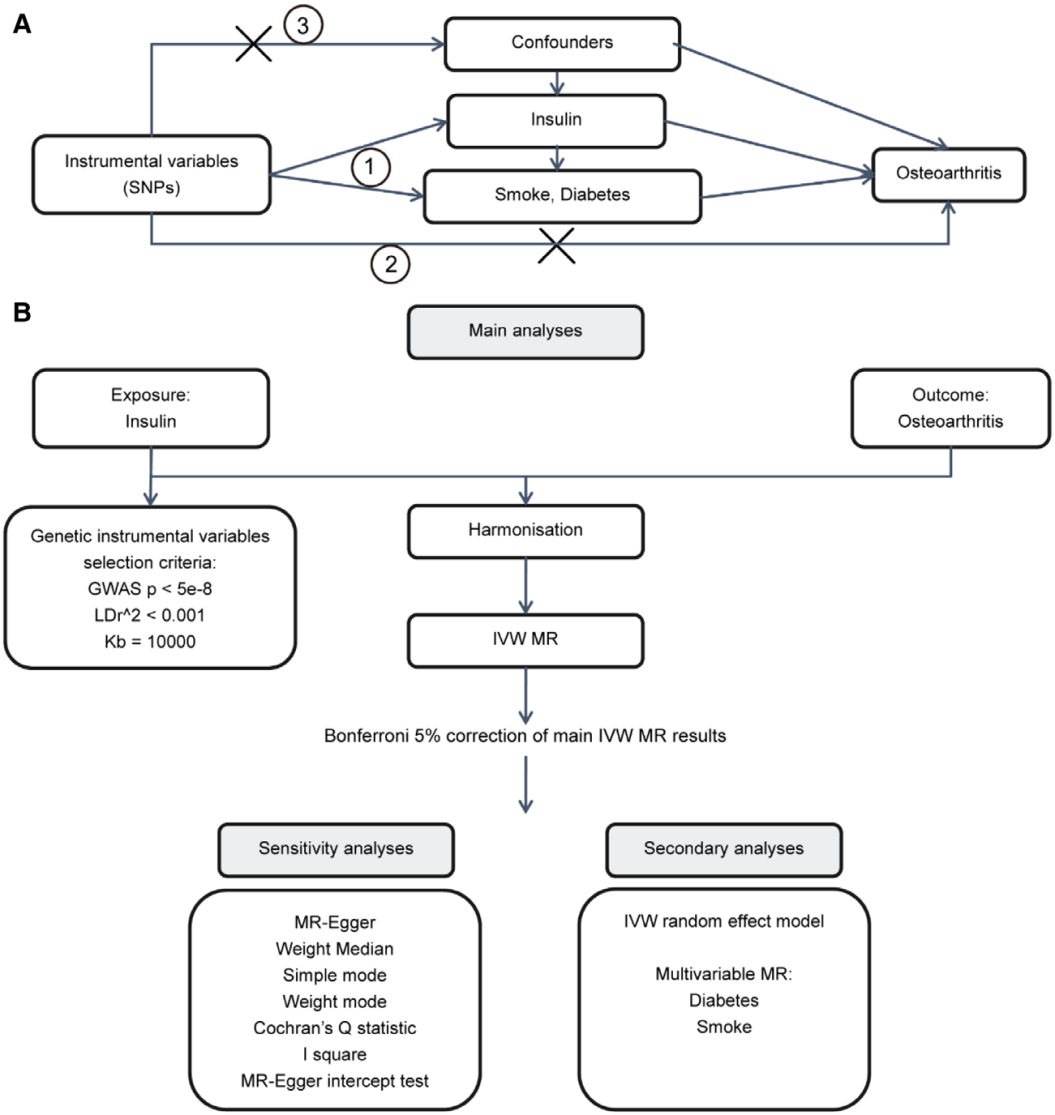
The digital technology roadmap (A) the basic assumptions of MR analysis, including (1) the association assumption that the selected instrumental variable must be significantly associated with the exposure; (2) exclusivity limitation, that is, the instrumental variable could only affect the outcome through the path of “instrumental variable → exposure → outcome”; and (3) independence assumption, that is, the instrumental variable must not be significantly related to potential confounders that might affect the exposure or outcome. (B) Flow chart of analysis methods in this study. GWAS = genome-wide association study, IVW = inverse variance weighted, LD = linkage disequilibrium, MR = Mendelian randomization, MR-Egger = Mendelian randomization-Egger, SNP = single nucleotide polymorphism.

A two-sample MR and a publicly available data set were used to investigate the causal relationship between exogenous insulin use and the risk of OA. And the mediating role of diabetes and smoking in the development of OA induced by exogenous insulin use. The research adhered to The Strengthening the Reporting of Observational Studies in Epidemiology Using MR (The STROBE-MR Statement) guidelines,^[14]^ and the schematic diagram is visually represented in Figure 1.

### 2.2. Data sources

We begin from the open genome-wide association study (GWAS) website (https://gwas.mrcieu.ac.uk/)^[15]^ GWAS data.

GWAS data for exogenous insulin use: GWAS data on exogenous insulin use (ukb-b-7350) was obtained by searching for “insulin” in the trait column of the downloaded GWAS data from males and females of the European race.

GWAS data for OA: We obtained GWAS data for OA (ieu-a-1169) by searching “osteoarthritis” in the trait column of the downloaded GWAS data. All GWAS data was obtained from European Males and Females samples.

Other GWAS data: risk factors associated with OA were obtained by reviewing the literature and corresponding GWAS data were obtained by downloading the GWAS data: diabetes^[16]^ (ebi-a-GCST90013891), and smoking (ieu-b-4857, ukb-b-2047). All GWAS data was derived from European samples.

### 2.3. Instrumental variable selection and strength assessment

A valid instrumental variable in genetic variation must fulfill 3 fundamental assumptions: the association hypothesis, indicating a significant relationship between the selected instrumental variable and the exposure factor; the independence assumption, ensuring that the instrumental variable is not significantly associated with potential confounders affecting the exposure or outcome; the exclusivity limitation, stipulating that the instrumental variable exclusively impacts the outcome through the path “instrumental variable → exposure → outcome.”

The screening criteria for Single Nucleotide Polymorphisms (SNPs) used as instrumental variables (IVs) in this study were as follows: SNPs with a *P*-value less than 5 × 10^−8^ in the exposure GWAS dataset were included for analysis. Additionally, SNPs in linkage disequilibrium, defined as SNPs with an *r*^2^ < 0.001 and a physical distance > 10,000 kb between every 2 genes, were excluded from the analysis. Outcome GWAS data was then extracted based on the selected SNPs. *F*-statistics were calculated to assess weak instrumental variable bias. When the *F* < 10, it signifies that the genetic variation utilized is a weak instrumental variable, which could potentially introduce bias into the results. Therefore, it is advisable to consider removing such variables from the analysis.^[17]^The formula for calculating the *F*-statistic is as follows:


$$\begin{array}{l} F=\frac{N-k-1}{k}\times \frac{R^{2}}{1-R^{2}} \end{array}$$

When N represents the sample size, k denotes the number of IVs utilized, and *R*^2^ indicates the degree to which the IVs account for the exposure. *R*^2^ = 2 × (1 − MAF) × MAF × ^2^β, where MAF is the minimum allele frequency and β is the allele effect size.

### 2.4. MR causal effect estimation

To evaluate the causal impact of exogenous insulin use on OA, several two-sample MR methodologies were employed, encompassing the instrumental variable weighting (IVW), MR-Egger, simple mode, weighted median method and weight mode (WM). The IVW method, a Wald estimate for each included SNP using meta-analysis, was our primary study method to estimate the overall effect of exposure on the outcome. In addition, WM and MR-Egger methods were used as additional tests for MR Estimation. The WM method is more effective when half of the genetic variants are invalid. The intercept of the MR-Egger method was used to assess the independence of instrumental variable strength from the direct effects hypothesis, which posits that horizontal pleiotropy effects are independent of the variable-exposure association. Horizontal pleiotropy was suggested by a *P*-value of <.05.

Researches have indicated^[18]^ that under certain conditions, the IVW method demonstrates a marginally superior robustness compared to other techniques; The distinct feature of the IVW approach lies in its disregard for the intercept term in the regression analysis, instead employing the inverse of the outcome variance as the weighting factor for estimation. Consequently, in scenarios devoid of pleiotropy, regardless of the presence or absence of heterogeneity, the IVW method is prioritized for main MR Analysis, with additional insights drawn from 4 other methods (employing the IVW random effects model in case of heterogeneity). Conversely, in the presence of pleiotropy, the MR-Egger method is utilized to ascertain the outcomes. Finally, we used the Steiger directivity test of the TwoSampleMR package to determine the direction of causality.

### 2.5. Sensitivity analysis

Sensitivity analysis was conducted through a series of tests, including tests for heterogeneity, pleiotropy, and individual exclusion, detailed as follows:

1. Heterogeneity test: The Cochran *Q* test was employed to assess the heterogeneity among SNP estimates. A statistically significant Cochran *Q* test indicated substantial heterogeneity in the analysis results. Although the Cochran *Q* test can determine the presence of heterogeneity, it does not evaluate the distribution of this heterogeneity. To quantify the extent of heterogeneity attributable to the IVs within the total variance, the I^2^ statistic was utilized. The formula for calculating I^2^ is designed to reflect the proportion of variance in the instrumental variable that is due to heterogeneity. *I*^2^ = 0 to 25% suggests mild heterogeneity, I^2^ = 25%-50% indicates moderate heterogeneity, and *I*^2^ > 50% signifies high heterogeneity. The *I*^2^ was adjusted to 0 (indicating no observed heterogeneity) when *I*^2^ is less than or equal to 0. The *I*^2^ values are interpreted as follows:

$$\begin{array}{l} I^{2}=\frac{Q-df}{Q}\times 100\% \end{array}$$

1. Pleiotropy test: The MR-Egger method is employed specifically to assess the pleiotropy of IVs in MR studies. This method involves testing the intercept from the MR-Egger regression, which serves as an indicator of the presence of horizontal pleiotropy among the genetic variants used as IVs. If the *P*-value associated with MR-Egger intercept is below .05, this is interpreted as evidence of significant horizontal pleiotropy.
2. Leave-one-out test: MR assesses the influence of each SNP on the link between exogenous insulin and OA by omitting each SNP sequentially. A significant change in MR effect estimates upon SNP exclusion indicates sensitivity to that specific SNP, suggesting its unique impact on the observed association.

### 2.6. Multivariate MR (MVMR) analysis and mediating effect estimation

MVMR expands on traditional MR by leveraging genetic variants linked to several related exposures to discern their collective impact on a singular outcome. This approach enables the assessment of the direct effect of each individual exposure on the outcome, isolating its contribution from other correlated exposures. We evaluated the effects of multiple associated risk factors for OA (diabetes, ebi-a-GCST90013891) and smoking (Smoke, ieu-b-4857; ukb-b-2047) and screened Insulin use (ukb-b-7350) were used together for MVMR Analysis to obtain the direct effect of exogenous insulin use on OA. In addition, risk factors associated with OA (diabetes and smoking) were used as mediators in subsequent analyses.

We obtained the effect of exogenous insulin use on diabetes and smoking by univariate MR to obtain the indirect effect of exogenous insulin use → diabetes and smoking → OA pathway (Fig. 1). Effect sizes and standard errors for mediating effects were calculated according to the following equation:


$$\begin{array}{l} \beta_{M}=\beta_{A}\times \beta_{B} \end{array}$$

$$\begin{array}{l} SE_{M}=\sqrt{{\left(\beta_{A}\times SE_{B}\right)}^{2}+{\left(\beta_{B}\times SE_{A}\right)}^{2}+S{E_{A}}^{2}\times S{E_{B}}^{2}} \end{array}$$

Where β_M_ is the effect value of mediating effect, β_A_ is the MR Effect value of exogenous insulin use on diabetes and smoking, β_B_ is the direct effect value of diabetes and smoking on OA (obtained by MVMR), SE_M_ is the standard error of mediating effect, SE_A_ is the MR Analysis standard error of exogenous insulin use on diabetes and smoking. SE_B_ is the standard error of the MR Analysis of diabetes and smoking on OA.

Combined with the causal stepwise regression method, if β_A_ and β_B_ are both significant, the indirect effect is significant. If β_A_ and β_B_ were not significant, Sobel test was used to determine whether β_M_ was significant. If β_M_ was significant, the indirect effect was significant. On the premise of indirect effect significantly, if the β_M_ and the use of exogenous insulin in MVMR on OA of the MR effect value β_C’_ opposite sign, is the use of exogenous insulin by diabetes and smoking OA of the mediation role of direct effect ratio calculation formula is: | β_M_/β_C’_ | × 100%. Exogenous insulin in the β_M_ and MVMR used for OA of the MR effect value of β_C’_ with number, is the use of exogenous insulin by diabetes and smoking OA the mediating role of the proportion of the total effect calculation formula is: β_M_/β_C_ by 100%, the β_C_ for the use of exogenous insulin in single variable MR effect value of OA.

### 2.7. Statistical analysis

Data computations and statistical evaluations were conducted using the R programming language (https://www.r-project.org/, version 4.2.2). For MR analysis The TwoSampleMR package was used.^[19]^ To assess the robustness and reliability of findings, the Cochran *Q* test and leave-one-out analysis were employed. The MR-Egger intercept method was applied to investigate genetic pleiotropy, while the Steiger directionality test, available within the TwoSampleMR package, was used to ascertain the causal direction between exposure and outcome. The metrics for evaluating the MR analysis were the odds ratio (OR) and the 95% confidence interval (95% CI). All statistical tests were two-sided, with a *P*-value below .05 deemed to indicate statistical significance.

## 3. Results

### 3.1. Technology roadmap

The technology roadmap is systematically delineated in Figure 1.

### 3.2. Instrumental variable screening

According to the criteria of IVs in this study, SNPS with linkage disequilibrium were removed. After matching the GWAS data of OA (ieu-a-1169), SNPS associated with the use of exogenous insulin (ukb-b-7350) were included as IVs. The results of instrumental variable screening for each indicator are shown in Table 1, and only the indicators that were significant by MR Analysis are shown in the table. The *F*-test statistic of the IVs of this index was >10, indicating that most of the SNPs screened in this study were strong-effect IVs, and the possible bias caused by weak IVs was limited.

**Table 1:** Screening of IVs and *F*-test of instrumental variable strength for the use of exogenous insulin and osteoarthritis.

Exposure	Number of SNPs	Median of *F*	Minimum of *F*	Maximum of *F*
ukb-b-7350	4	78.13578436	56.23444889	169.9527

SNP = single nucleotide polymorphism.

### 3.3. Estimation of MR causal effects

In the analysis employing various models such as MR-Egger, weighted median, IVW, simple mode, and WM, the IVW model’s findings highlighted that the use of exogenous Insulin (ukb-b-7350, OR_IVW_ = 0.00029, 95% CI: 0–0.694, *P*_IVW_ = .040158) had a significant causal association with OA (ieu-a-1169) (Table 2).

**Table 2:** Results of the Mendelian randomization analysis of the effect of exogenous insulin on the incidence of osteoarthritis.

Exposure	Number of SNPs	OR	95% CI	*P*-value
ukb-b-7350	4	0.00029	0–0.694	.040158

CI = confidence interval, MR = Mendelian randomization, OR = odds ratio, SNP = single nucleotide polymorphism, β = effect coefficient of MR analysis.

**Figure 2.**
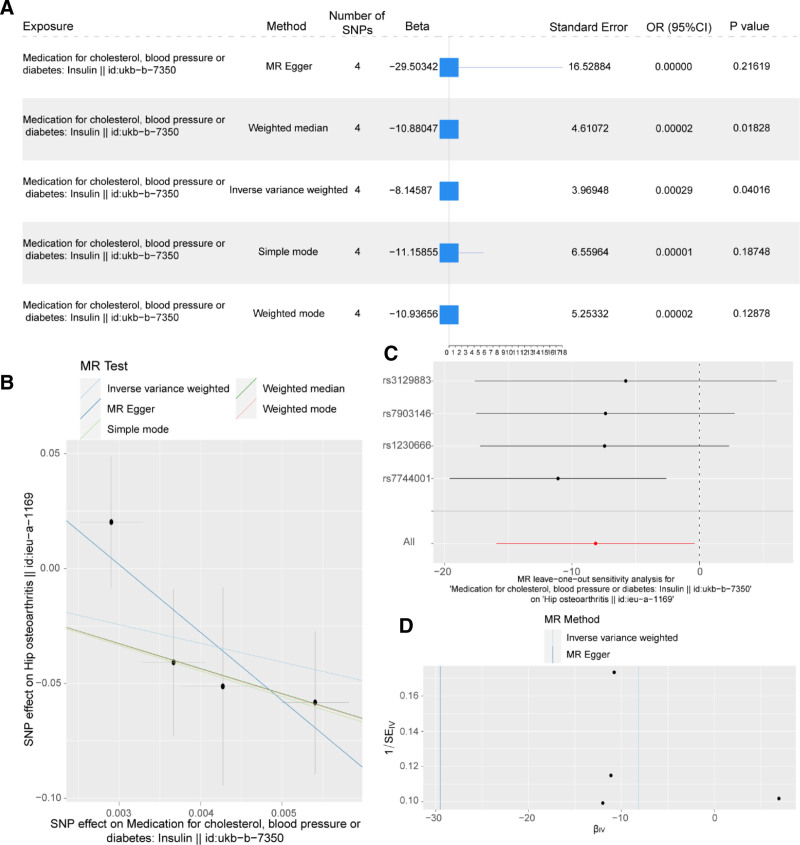
Mendelian randomization analysis of the causal relationship between exogenous insulin use and Osteoarthritis. (A) Forest plot showing the results of the MR analysis of the effect of exogenous insulin use on the occurrence of OA. (B) Scatter plot of the results of MR analysis of the causal relationship between exogenous insulin use and OA. (C) Forest plot of the results of MR analysis of the causal relationship between exogenous insulin use and OA. (D) Funnel plot showing the results of MR analysis between exogenous insulin use and OA. CI = confidence interval, MR = Mendelian randomization, OA = osteoarthritis, OR = odds ratio, SNP = single nucleotide polymorphism.

In addition, the results of the MR analysis of Insulin (ukb-b-7350) and OA (ieu-a-1169) are presented as forest plots (Fig. 2A). Figure 2B shows the linear relationship between the effect of the IVs on the use of Insulin (ukb-b-7350) and the effect on the occurrence of OA (ieu-a-1169) under the 5 models. The direction of effect size estimated by MR-Egger, weighted median, simple mode, and WM was consistent with that of the IVW model. The slope represents the value of β, that is, the strength of linear dependence among the instrumental variable, the effect of exposure, and the effect on the outcome.

The Cochran *Q* test was utilized to evaluate the heterogeneity in the results of the MR-Egger and IVW models, finding no significant heterogeneity in the MR outcomes related to OA and the use of exogenous insulin (Cochran *Q P*-value > .05) (Table S1, Supplemental Digital Content, https://links.lww.com/MD/Q678). Additionally, a leave-one-out analysis was conducted to assess the impact of each instrumental variable locus on the causal effect of OA (ieu-a-1169) by sequentially removing each one (Fig. 2C). The funnel plot (Fig. 2D) for the instrumental variable concerning insulin use (ukb-b-7350) displayed a symmetrical distribution of causal effects, which points to the absence of potential bias in the results.

Following the initial analysis, MR-Egger regression was applied to assess the presence of horizontal pleiotropy among the IVs. The *P*-value for the intercept term related to insulin (ukb-b-7350) exceeded .05, and the intercept itself approximated zero. This outcome suggests that the causal relationship identified in this study between insulin use and OA is unlikely to be confounded by horizontal pleiotropy (Table 3).

**Table 3:** Tests of horizontal pleiotropy of the Mendelian randomization analysis of the use of exogenous insulin on the onset of osteoarthritis.

Exposure	MR-Egger intercept	Standard error	*P*-value
Medication for cholesterol, blood pressure or diabetes: Insulin	0.090171	0.0677	.3146

MR = Mendelian randomization.

Finally, the Steiger directivity test was used to determine whether the causal direction of the use of exogenous insulin (ukb-b-7350) to OA (ieu-a-1169) was correct (Table 4). The Steiger directional test calculated the variance explanation rate (*r*^2^) of SNPS for exposure and outcome, respectively. The results showed that the SNP of our selected index explained more variance for exposure than for outcome, with the direction of TRUE and *P* < .05, indicating that the direction was significantly correct.

**Table 4:** Mendelian randomization analysis of the use of exogenous insulin for Osteoarthritis Steiger directivity test.

Exposure	Outcome	SNP *r*^2^ exposure	SNP *r*^2^ outcome	Correct causal direction	Steiger *P*-value
ukb-b-7350	ieu-a-1169	0.001824	0.000525	TRUE	.021954

*r*^2^ = variance explained rate, SNP = single nucleotide polymorphism.

### 3.4. Reverse MR analysis

For the OA (ieu-a-1169) GWAS, an initial filtering was performed to identify SNPs exhibiting a *P*-value less than 5 × 10^−8^. Subsequently, SNPs in linkage disequilibrium were excluded based on criteria including an LD-*r*^2^ value <0.001 and a physical distance >10,000 kb between pairs of genes. The analysis findings indicated a lack of significant causal association between OA (ieu-a-1169) and any major exposure factors under consideration. Furthermore, it was determined that the occurrence of OA (ieu-a-1169) had a minimal likelihood of influencing the usage of exogenous insulin (ukb-b-7350).

**Figure 3.**
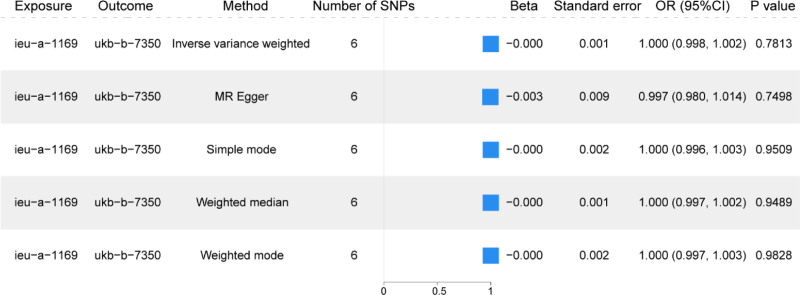
Mendelian randomization analysis of the causal relationship between osteoarthritis and exogenous insulin use. Forest plot of the results of the MR analysis of the incidence of OA (ieu-a-1169) on the use of exogenous insulin (ukb-b-7350). CI = confidence interval, OA = osteoarthritis, MR = Mendelian randomization, OR = odds ratio, SNP = single nucleotide polymorphism.

In a subsequent analysis phase, SNPs with a *P*-value less than 5 × 10^−6^ were identified and further filtered to exclude those in linkage disequilibrium, adhering to the criteria of an LD-*r*^2^ value below 0.001 and a physical distance exceeding 10,000 kb between each pair of genes. The MR analysis utilized a comprehensive suite of models including the MR-Egger model, weighted median, IVW model, Simple mode model, and WM, to assess the SNPS. Consistent with previous findings, IVW model results also reaffirmed no significant causal relationship between OA (ieu-a-1169) and exogenous insulin (ukb-b-7350), and OA (ieu-a-1169) were less likely to cause changes in the use of exogenous insulin (Table S2, Supplemental Digital Content, https://links.lww.com/MD/Q678). OA (ieu-a-1169) and exogenous insulin use (ukb-b-7350) were also presented as forest plots (Fig. 3).

### 3.5. Multivariable MR analysis of the use of exogenous insulin on the onset of osteoarthritis

We incorporated related risk factors for OA (diabetes [ebi-a-GCST90013891] and smoking [ieu-b-4857, ukb-b-2047]) along with the use of exogenous insulin (ukb-b-7350) in a multivariable MR analysis to evaluate the direct effect of exogenous insulin use (ukb-b-7350) on OA (ieu-a-1169).

Two OA risk factors (diabetes, smoke) and insulin (ukb-b-7350) were separately used to construct models to predict the relationship between the 2 risk factors and the outcomes. Three significant multivariate MR Models were obtained (Table 5). The results showed that model 1 took diabetes (ebi-a-GCST90013891) and insulin (ukb-b-7350) as exposure, and the results showed that after removing the effect of diabetes, the use of exogenous insulin (ukb-b-7350) was significantly higher than that of exogenous insulin (ukb-b-7350) still had a direct effect on OA (ieu-a-1169) (*P* < .05). Model 2 used Smoke (ieu-b-4857) and exogenous insulin (ukb-b-7350) as exposures, and the results showed that after removing the effect of Smoke, the exogenous insulin use (ukb-b-7350) was significantly higher than that of the control group (*P* < .05). Exogenous insulin (ukb-b-7350) still had a direct effect on OA (ieu-a-1169) (*P* < .05). Model 3 used smoking (ukb-b-2047) and exogenous insulin (ukb-b-7350) as exposures, and the results showed that exogenous insulin use (ukb-b-7350) after removing the effect of Smoke still had a direct effect on OA (ieu-a-1169) (*P* < .05).

**Table 5:** Multivariable Mendelian randomization analysis of the use of exogenous Insulin (ukb-b-7350) on the incidence of Osteoarthritis.

Model	Exposure	Number of SNPs	OR (95% CI)	*P*-value
Model1	ebi-a-GCST90013891	1	1.112113	.029543
	ukb-b-7350	3	3.8 × 10^−8^	.006729
Model2	ieu-b-4857	0	3.74 × 10^11^	4.65 × 10^−16^
	ukb-b-7350	3	2.38 × 10^−17^	2.17 × 10^−20^
Model3	ukb-b-2047	0	0.018599	0
	ukb-b-7350	3	3.28 × 10^−5^	0

CI = confidence interval, MR = Mendelian randomization, OR = odds ratio, SNP = single nucleotide polymorphism.

### 3.6. Mediation effect analysis

First, risk factors related to OA (diabetes, smoke) were used as mediators in the subsequent analysis. We then evaluated the mediation effect of models in which the mediator had a significant causal relationship with the outcome in the multivariate MR Analysis. In model 1, the use of exogenous insulin (ukb-b-7350) had a significant effect on OA (*P* < .05), and it could be determined that the use of exogenous insulin (ukb-b-7350) affected OA through diabetes (ebi-a-GCST90013891), which formed a partial mediating effect model. In model 2, the use of exogenous insulin (ukb-b-7350) had a significant effect on OA (*P* < .05), which could be determined that the use of exogenous insulin (ukb-b-7350) affected OA through smoking (ieu-b-4857), forming a partial mediating effect model. In model 3, the use of exogenous insulin (ukb-b-7350) had a significant effect on OA (*P* < .05), which could be determined that the use of exogenous insulin (ukb-b-7350) affected OA through smoking (ukb-b-2047), forming a partial mediating effect model.

The causal effect of exogenous insulin use (ukb-b-7350) on diabetes and smoking was obtained from univariate MR Analysis, and the direct effect of diabetes and smoking on OA was obtained from multivariate MR Analysis. The mediating effect of exogenous insulin on OA through diabetes and smoking was calculated. The results are shown in Table 6. Results show that the use of exogenous insulin (ukb-b-7350) through diabetes (ebi-a-GCST90013891) for OA of the intermediary effect is 12.68639, the mediation effect of 74.26% (12.68639/ −17.08472 | | * 100%); The use of exogenous insulin (ukb-b-7350) by smoking (ieu-b-4857) for the mediation effect of OA is 26.83313, the mediation effect of 70.11% (26.83313/ −38.27482 | | * 100%); The use of exogenous insulin (ukb-b-7350) by smoking (ukb-b-2047) for the mediation effect of OA is 4.4753, the mediation effect of 43.34% (4.4753/ −10.32532 | | * 100%).

**Table 6:** Diabetes and smoking mediated the use of exogenous insulin (ukb-b-7350) in osteoarthritis (ieu-a-1169) Mendelian randomization mediating role in the analysis of evaluation.

Exposure	Mediator	Outcomes	Total effect (95% CI)	Effect E-M (95% CI)	Effect M-O (95% CI)	Effect E-O (95% CI)	Mediation effect (95% CI)
ukb-b-7350	ebi-a-GCST 90013891	Hip osteoarthritis	−8.14587 (−15.92605, −0.36569)	119.39012 (93.16793, 145.61231)	0.10626 (0.01055, 0.20197)	−17.08472 (−29.44127, −4.72817)	12.68639 (0.92512, 24.44766)
ukb-b-7350	ieu-b-4857	Hip osteoarthritis	−8.14587 (−15.92605, −0.36569)	1.00701 (0.12029, 1.89373)	26.64634 (20.21472, 33.07796)	−38.27482 (−46.38195, −30.16769)	26.83313 (2.3336, 51.33266)
ukb-b-7350	ukb-b-2047	Hip osteoarthritis	−8.14587 (−15.92605, −0.36569)	−1.12314 (−2.23681, −0.00947)	−3.98463 (−4.07628, −3.89298)	−10.32532 (−10.54802, −10.10262)	4.4753 (0.03653, 8.91407)

Total effect: the effect of exposures on outcomes; Effect E-M: the effect of exposures on mediators; Effect M-O: the effect of mediators on outcomes; Effect E-O: the direct effect after considering the intermediary effect; Mediation effect: the indirect effect of exposures on outcomes via mediators.

CI = confidence interval.

## 4. Discussion

Following adjustments for instrumental variable strength and heterogeneity, our application of diverse MR models, including the IVW model, revealed a notable causal link between exogenous insulin usage and reduced OA risk (OR_IVW = 0.00029, 95% CI: 0 to 0.694, *P*_IVW = 0.040158). This finding is supported by existing literature. For instance, Al-Jarallah et al observed that insulin therapy in DM patients was linked to diminished radiographic osteophyte formation in knee OA, suggesting insulin’s potential protective role against OA progression in this demographic.^[20]^ Similarly, Nieves-Plaza et al found that, even after adjusting for variables like age, obesity, depression, and cardiovascular complications, the lack of insulin use was significantly correlated with a heightened risk of hand or knee OA. Specifically, DM patients not treated with insulin faced a 4.44-fold increased risk of developing OA.^[21]^ Contrastingly, other research posits that insulin may exacerbate OA by fostering the synthesis of pro-inflammatory agents such as interleukins, TNF-alpha, and MMP-13, which are implicated in OA’s pathogenesis.^[22,23]^ Several reasons may explain these divergent findings. Primarily, our study’s reliance on genetic variants as IVs allows for a causal inference with minimized confounding, a methodological advantage not present in observational studies. Such differences in approach can yield varying outcomes due to the distinct handling of confounders. Furthermore, the pathophysiology of OA, particularly within the context of DM and insulin therapy, is inherently complex. Insulin exhibits both anabolic and catabolic effects on musculoskeletal tissues, effects that likely vary among patient populations and disease stages.

Our results showed that exogenous insulin (ukb-b-7350) mediated OA through diabetes (ebi-a-GCST90013891) with a mediation effect of 74.26% (12.68639/ −17.08472 | | * 100%). diabetes as a very important risk factor influences the development and progression of OA, recent studies increasingly report a relationship between diabetes and OA. In a meta-analysis focused on studies that accounted for weight or Body Mass Index (BMI), the prevalence of OA among patients with DM remained significantly higher compared to the non-DM population, exhibiting an OR of 1.25.^[24]^ This finding is further supported by Eymard et al, who reported that individuals with DM witnessed a more rapid progression of knee OA over a 3-year period than those without diabetes.^[25]^

Exogenous insulin, introduced externally, plays a pivotal role in managing blood glucose levels in individuals with insufficient or absent natural insulin production, commonly due to DM. DM accelerates OA progression through 2 primary mechanisms: hyperglycemia and insulin resistance.^[26]^ Hyperglycemia impedes the chondrogenic differentiation of mesenchymal,^[27]^ muscle,^[28]^ and adipose-derived stem cells,^[29]^ undermining the limited cartilage regeneration capacity inherent in OA. Additionally, it disrupts chondrocyte metabolism, evidenced by increased IL-6 and PGE2 production and reduced autophagy and heme oxygenase expression under IL1-beta stimulation in diabetic cartilage, signaling the suppression of critical antioxidant pathways.^[30–32]^ Insulin resistance exacerbates joint tissue damage, stemming from both localized diabetic synovial membrane resistance^[33]^ and systemic low-grade inflammation.^[34]^ Notably, the synovium in obese OA and DM patients shows marked insulin resistance,^[35]^ diminishing insulin’s capacity to mitigate synovial inflammation and catabolism. Consequently, insulin resistance in obesity hampers the efficacy of insulin in restraining the release of inflammatory and catabolic factors, furthering OA’s progression.^[33]^ However, exogenous insulin and oral anti-diabetic medications exhibit pleiotropic effects, including anti-inflammatory properties^[36,37]^ and cartilage protection,^[38,39]^ offering therapeutic avenues against OA’s advancement.

This MR study explores the causal link between the use of exogenous insulin and the risk of OA, while also examining the intermediary roles of diabetes and smoking. One of the primary strengths of this investigation is its innovative use of genetic variants as IVs, significantly reducing the confounding factors often encountered in observational studies. This strategy bolsters the reliability of our causal conclusions, mirroring the methodological rigor of a randomized controlled trial. By employing a diverse array of MR analytical techniques, our study enhances the robustness and credibility of the findings. Furthermore, the use of publicly accessible GWAS datasets not only fosters transparency but also facilitates the reproducibility and external validation of our results. Ultimately, this research contributes novel scientific insights into the management of diabetes and OA, advocating for integrated treatment approaches.

Recognizing the limitations of this MR study is essential for a comprehensive interpretation of its findings. Although the 4 SNPs selected in this study all exceeded the conventional weak instrument threshold (*F* > 10) in terms of *F*-statistics, theoretically ensuring strong individual instrument strength, the limited number of IVs introduces several important limitations. First, the small number of IVs may reduce the overall statistical power of the analysis and increase the sensitivity of results to the influence of individual SNPs. When the number of IVs is limited, the ability to detect and correct for horizontal pleiotropy and weak instrument bias diminishes, potentially compromising the robustness of causal inference. Second, the extremely low odds ratio (OR = 0.00029) observed in this study, while statistically significant, should be interpreted with caution in terms of biological plausibility. This may partly stem from estimation instability or residual confounding due to the limited number of IVs. Finally, the restricted set of IVs also constrains our ability to conduct more comprehensive sensitivity and subgroup analyses. Therefore, while the findings provide preliminary evidence for a causal relationship between exogenous insulin use and osteoarthritis risk, we recommend that future studies incorporate larger-scale GWAS data to identify additional independent and robust SNPs as IVs, thereby further validating and refining these findings.

## 5. Conclusion

This MR study establishes a causal connection between the use of insulin and an elevated risk of OA, with diabetes and smoking acting as significant mediators. These findings highlight the critical need for comprehensive management strategies in diabetic patients, aiming to optimize the therapeutic benefits of insulin while mitigating its potential risks related to OA. By doing so, this research paves the way for future targeted interventions, offering valuable insights for the development of more nuanced treatment plans that consider both the glycemic control and joint health of patients with diabetes.

## Acknowledgments

We extend our gratitude to the United Kingdom Biobank and the IEU Open GWAS Project for making their summary statistics publicly available for use in our study.

## Author contributions

**Conceptualization:** Jichao Liu.

**Data curation:** Jinzhe Zheng.

**Funding acquisition:** Wei Li, Gensheng Zhang, Yangyang Liu, Jichao Liu.

**Methodology:** Jinzhe Zheng.

**Software:** Jinzhe Zheng.

**Writing – original draft:** Jinzhe Zheng.

**Writing – review & editing:** Wei Li, Gensheng Zhang, Yangyang Liu, Jichao Liu.

## Supplementary Material
